# Accuracy of two deep learning–based reconstruction methods compared with an adaptive statistical iterative reconstruction method for solid and ground-glass nodule volumetry on low-dose and ultra–low-dose chest computed tomography: A phantom study

**DOI:** 10.1371/journal.pone.0270122

**Published:** 2022-06-23

**Authors:** Cherry Kim, Thomas Kwack, Wooil Kim, Jaehyung Cha, Zepa Yang, Hwan Seok Yong

**Affiliations:** 1 Department of Radiology, Ansan Hospital, Korea University College of Medicine, Ansan-si, Gyeonggi, South Korea; 2 Department of Radiology and Medical Imaging, University of Virginia Health System, Charlottesville, VA, United States of America; 3 Medical Science Research Center, Ansan Hospital, Korea University College of Medicine, Ansan-si, Gyeonggi, South Korea; 4 Biomedical Research Center, Guro Hospital, Korea University College of Medicine, Seoul, South Korea; 5 Department of Radiology, Guro Hospital, Korea University College of Medicine, Seoul, South Korea; Pisa University Hospital, ITALY

## Abstract

No published studies have evaluated the accuracy of volumetric measurement of solid nodules and ground-glass nodules on low-dose or ultra–low-dose chest computed tomography, reconstructed using deep learning–based algorithms. This is an important issue in lung cancer screening. Our study aimed to investigate the accuracy of semiautomatic volume measurement of solid nodules and ground-glass nodules, using two deep learning–based image reconstruction algorithms (Truefidelity and ClariCT.AI), compared with iterative reconstruction (ASiR-V) in low-dose and ultra–low-dose settings. We performed computed tomography scans of solid nodules and ground-glass nodules of different diameters placed in a phantom at four radiation doses (120 kVp/220 mA, 120 kVp/90 mA, 120 kVp/40 mA, and 80 kVp/40 mA). Each scan was reconstructed using Truefidelity, ClariCT.AI, and ASiR-V. The solid nodule and ground-glass nodule volumes were measured semiautomatically. The gold-standard volumes could be calculated using the diameter since all nodule phantoms are perfectly spherical. Subsequently, absolute percentage measurement errors of the measured volumes were calculated. Image noise was also calculated. Across all nodules at all dose settings, the absolute percentage measurement errors of Truefidelity and ClariCT.AI were less than 11%; they were significantly lower with Truefidelity or ClariCT.AI than with ASiR-V (all P<0.05). The absolute percentage measurement errors for the smallest solid nodule (3 mm) reconstructed by Truefidelity or ClariCT.AI at all dose settings were significantly lower than those of this nodule reconstructed by ASiR-V (all P<0.05). Furthermore, the lowest absolute percentage measurement errors for ground-glass nodules were observed with Truefidelity or ClariCT.AI at all dose settings. The absolute percentage measurement errors for ground-glass nodules reconstructed with Truefidelity at ultra–low-dose settings were significantly lower than those of all sizes of ground-glass nodules reconstructed with ASiR-V (all P<0.05). Image noise was lowest with Truefidelity (all P<0.05). In conclusion, the deep learning–based algorithms were more accurate for volume measurements of both solid nodules and ground-glass nodules than ASiR-V at both low-dose and ultra–low-dose settings.

## Introduction

Since the National Lung Cancer Screening Trial (NLST) emphasized the need for low-dose chest computed tomography (CT) screening to reduce mortality from lung cancer in 2011 [1], the demand for lung cancer screening with low-dose chest CT has been increasing. However, measures to minimize CT-associated radiation exposure are of particular importance for lung cancer patients, given the frequency with which follow-up CT scans are performed [2]. Therefore, iterative reconstruction (IR) has been a good option for minimizing radiation exposure while optimizing the quality of low-dose chest CT images for nodule volumetry [3–5].

Recently, several studies have revealed improved image quality—for all organs and body parts—achieved with deep learning–based image reconstruction relative to that achieved with IR of CT scans [6–18]. These studies have shown deep learning–based reconstruction (TrueFidelity [TFI]) to be superior to IR (adaptive statistical IR, ASiR-V) in terms of image noise and sharpness [6, 11]. Additionally, deep learning–based reconstruction has been associated with higher signal-to-noise ratio (SNR) and contrast-to-noise ratio (CNR) values than ASiR-V with low-dose chest CT [11].

However, no published studies have evaluated the accuracy of volumetric measurement of solid nodules (SNs) and ground-glass nodules (GGNs) on low-dose or ultra–low-dose chest CT, reconstructed using deep learning–based algorithms. This is an important issue in lung cancer screening. Automatic or semiautomatic volumetric assessment of lung nodules is known to be an accurate method for providing consistent and reproducible measurements superior to those obtained using nonvolumetric and manual techniques [2, 19]. However, the improved image quality or sharpness achieved with deep learning–based reconstruction may not directly reflect the accuracy of lung nodule volumetry, and studies on the accuracy of volumetric measurements made using low-dose or ultra–low-dose CT scans reconstructed by deep learning–based algorithms are needed.

Additionally, although TFI has been associated with higher image quality than IR, TFI can only be applied to images obtained using a specific CT scanner and cannot be applied using low-dose CT images obtained using other CT devices [6]. In contrast, ClariCT.AI (ClariPI Inc.) is a deep learning–based denoising solution that is compatible with any CT scanner.

This study aimed to compare the accuracy of two deep learning–based algorithms (TFI and ClariCT.AI) with IR (ASiR-V) for volume measurement of SNs and GGNs of various sizes at low-dose and ultra–low-dose settings.

## Materials and methods

This study did not require institutional review board approval because it did not involve any human or animal subjects.

### Mimicking the human thorax: Anthropomorphic thoracic phantom with synthetic lung nodules

A commercial multipurpose anthropomorphic chest phantom (Lungman; Kyoto Kagaku Co., Ltd, Kyoto, Japan) was used to mimic the human thorax. This phantom is a life-sized anatomical human male thorax model consisting of soft tissue substitute materials and synthetic bones, all of which show X-ray attenuation properties similar to their corresponding human tissues. Three-dimensional synthetic pulmonary vessels and bronchi were also inserted into the phantom for structural similarity.

In total, ten spherical synthetic pulmonary nodules were used, and the characteristics of those nodules are described in Table 1: four different-sized SNs (3 mm, 5 mm, 8 mm, and 10 mm in diameter) and three different-sized GGNs (5 mm, 8 mm, and 10 mm in diameter). The attenuation values of the GGNs were −630 and −800 Hounsfield units (HU). Since all nodule phantoms are perfectly spherical, the volume can be calculated using the diameter. The nodules were randomly placed and fixed within in the phantom using double-sided tape.

**Table 1:** Characteristics of ten spherical synthetic nodules.

Nodule types	Diameter (mm)	Reference volume (mm^3^)	Density (HU)
SN	3	14.1	100
	5	65.5	
	8	268.1	
	10	523.6	
GGN	5	65.5	−630
	8	268.1	
	10	523.6	
	5	65.5	−800
	8	268.1	
	10	523.6	

Note—SN, solid nodule; GGN, ground-glass nodule.

### CT image acquisition

All CT images were obtained using a Revolution ES scanner (GE Healthcare, Chicago, IL, USA). Image acquisition was carried out using four different radiation doses (120 kVp/220 mA, 120 kVp/90 mA, 120 kVp/40 mA, and 80 kVp/40 mA). The study protocol and radiation dose data are summarized in Table 2. The CT dose index volume (CTDI_vol_) and dose length product (DLP) were recorded for all CT examinations, and the effective dose (ED) was calculated using a conversion coefficient (0.014 mSv/mGy×cm) for chest CT [20]. The reconstruction algorithm was “Lung”. The scan parameters were as follows: noise index, 15; gantry rotation time, 0.35 s; coverage speed, 350 mm/s; pitch, 1.53:1; and slice thickness, 1.25 mm.

**Table 2:** Summary of the study protocol and radiation dose data.

Radiation dose	CT dose index volume (mGy)	Dose length product (mGy×cm)	Effective dose (mSv)*
120 kVp/220 mA	3.39	147.56	2.07
120 kVp/90 mA	1.39	60.37	0.85
120 kVp/40 mA	0.62	26.83	0.38
80 kVp/40 mA	0.2	8.49	0.12

*Tissue weighting factor for calculating the effective dose: 0.014 mSv/mGy×cm

### Image reconstruction algorithms

Ten nodules scanned in four different radiation dose settings were reconstructed with ASiR-V with a blending factor of 70%, TFI with a high strength level, and ClariCT.AI. Overall, three different reconstruction algorithms (ASiR-V, TFI, and ClariCT.AI) were used with each dose setting. Therefore, a total of 120 reconstruction imaging datasets were finally obtained and analyzed.

### Nodule volume and image noise measurements

All nodule volumes were measured by two radiologists (T.K. and C.K., with 1 and 11 years of experience in thoracic imaging, respectively, to determine the interobserver variability) using commercially available software (Aquarius iNtuition Edition, Terarecon, Foster City, CA, USA) previously used for nodule volumetry in several studies [3, 21, 22].

Semiautomatic nodule segmentation was performed by clicking at the center of each nodule. The default segmentation attenuation thresholds representing SNs and GGNs were −850 HU and −300 HU, respectively. Further adjustments of these thresholds were performed by the radiologists if the software-based nodule segmentation was determined to be inadequate in consensus. However, most segmentation procedures were performed with a single click without manual modification. These measurements were repeated four times.

The absolute percentage measurement error (APE), which is the difference between the measured volume and the reference volume, was calculated for analysis, as described previously [3, 22]. The reference volume can be calculated using the diameter since all nodule phantoms are perfectly spherical. The APE of each nodule volume was calculated as follows: |measured nodule volume in each algorithm − reference nodule volume| × 100 / reference nodule volume. The volume of all nodules, regardless of size and type, was measured four times, and the APE value was calculated using the reference volume of each nodule from each measurement value. After that, the mean and standard deviation of the APE values of each nodule were calculated. Since the measurements were repeated, APEs are presented as mean ± standard deviation (SD). The results are presented in terms of nodule type (across all nodules, all SN, all GGN), in terms of both nodule size and nodule type, and in terms of two attenuation levels (-630 HU and -800 HU) for GGNs.

Image noise was also calculated to assess the image quality by averaging three different SDs of attenuation, as described previously [3]: two values were from both lung fields of the phantom (right posteromedial lung field near the mediastinum and left posterolateral lung field near the thoracic wall at the level of the heart), and one value was from the room air outside of the chest wall (3 cm away from the anteromedial chest wall). A circular region of interest (ROI) with an area of 120 mm^2^ was used.

### Task-based transfer function (TTF) and noise power spectrum (NPS)

TTF and NPS were also analyzed using an ACR (American College of Radiology) CT certified phantom (Gammex 464, Sun Nuclear, Middleton, WI, USA) to evaluate quantitative image quality at the different radiation dose levels. The phantom used in the experiment has a module composed of four layers, NPS was measured in module 3 with a homogeneous medium, and TTF was measured in module 1 including cylindrical inserts of various materials. In this experiment, TTF was measured using bone and acrylic cylinder rods. To quantify TTF, we calculated the spatial frequency (TTF_50%_) value at the point where the y-axis value becomes 0.5 in the measured TTF curve. Additionally, we directly implemented the 3D-based NPS used by many researchers based on the method presented by the American Association of Physicists in Medicine (AAPM) [23].

The TTF used imQuest (Duke University, Durham, NC, USA) software, implemented using Matlab (Version R2017a, The MathWorks, Inc., Natick, MA, USA), and the NPS of the ACR phantom image was implemented and calculated using Matlab.

### Statistical analysis

For the repeated measures data analysis, repeated measures analysis of variance (RM ANOVA) as a parametric test or Friedman’s test as a nonparametric test was performed. Bonferroni correction was used to adjust the significance level and confidence interval for multiple comparisons of main effects. When the sphericity assumption by Mauchly’s test of sphericity was not met, the P-value from the Greenhouse-Geisser correction was used for tests of within-subject effects. Intraclass correlation coefficient (ICC) analysis was used to evaluate interobserver variability. ICC results were interpreted as follows: <0.40, poor agreement; 0.40–0.59, fair agreement; 0.60–0.74, good agreement; and 0.75–1.00, excellent agreement. A P-value less than 0.05 was considered statistically significant. All statistical analyses were performed using SPSS Statistics for Windows, version 25 (IBM Corp., Armonk, NY, USA).

## Results

### Mean APE according to nodule type

The APEs for different nodule types are shown in Table 3. The mean APEs of TFI and ClariCT.AI across all nodules at all dose settings were less than 11%.

**Table 3:** Absolute percentage measurement error according to nodule type.

Radiation dose (CTDI_vol_)	Nodule type	ASiR-V	TFI	ClariCT.AI	P-value
120 kVp/220 mA (3.39 mGy)	Across all nodules	7.68 ± 7.12	4.12 ± 5.39*	2.67 ± 3.41*	**<0.001**
	SN	9.49 ± 7.69	8.79 ± 7.75	4.82 ± 1.09	0.081
	GGN	6.47 ± 1.41	1.02 ± 0.22*	1.23 ± 0.28*	**<0.001**
120 kVp/90 mA (1.39 mGy)	Across all nodules	11.96 ± 9.97	5.02 ± 4.51*	5.88 ± 4.27*	**0.002**
	SN	9.36 ± 8.28	8.18 ± 5.42	6.43 ± 4.50	0.340
	GGN	13.70 ± 10.78	2.93 ± 1.94*†	5.51 ± 4.16*	**<0.001**
120 kVp/40 mA (0.62 mGy)	Across all nodules	13.58 ± 7.08	4.78 ± 5.78*†	8.34 ± 6.78*	**<0.001**
	SN	9.91 ± 6.55	8.14 ± 7.47	6.20 ± 3.87*	0.087
	GGN	16.03 ± 6.44	2.54 ± 2.37*†	9.76 ± 7.93*	**<0.001**
80 kVp/40 mA (0.2 mGy)	Across all nodules	18.15 ± 9.72	7.39 ± 7.23*	7.75 ± 6.41*	**<0.001**
	SN	14.21 ± 10.91	10.50 ± 8.94*	7.86 ± 7.67*	**0.012**
	GGN	20.78 ± 8.04	5.31 ± 5.03*	7.69 ± 5.59*	**<0.001**

Note—CTDI_**vol**_, CT dose index volume, SN, solid nodule; GGN, ground-glass nodule.

“Across all nodules” refers to the mean APEs from all SNs with diameters of 3, 5, 8, and 10 mm and all GGNs with diameters of 5, 8, and 10 mm.

*Comparison with ASiR-V, P<0.05

†Comparison with ClariCT.AI, P<0.05

Across all nodules at all dose settings, the APEs were significantly lower with the deep learning–based algorithms, TFI or ClariCT.AI, than with ASiR-V (all P<0.05). At 120 kVp/90 mA (CTDI_vol_, 1.39 mGy), 120 kVp/40 mA (CTDI_vol_, 0.62 mGy), and 80 kVp/40 mA (CTDI_vol_, 0.2 mGy), TFI yielded the lowest APE among the algorithms and a significantly lower APE than ASiR-V (all P<0.05). TFI also yielded a significantly lower APE than ClariCT.AI at 120 kVp/40 mA (CTDI_vol_, 0.62 mGy) (P = 0.01). At 120 kVp/220 mA, ClariCT.AI yielded the lowest APE among the algorithms (P<0.001).

The APEs of GGNs were significantly lower with the deep learning–based algorithms, TFI or ClariCT.AI, than with ASiR-V (all P<0.05) at all dose settings. Additionally, TFI yielded the lowest APE among the algorithms a significantly lower APE than ASiR-V (all P<0.05) at all dose settings, and a significantly lower APE than ClariCT.AI at 120 kVp/90 mA (CTDI_vol_, 1.39 mGy) and 120 kVp/40 mA (CTDI_vol_, 0.62 mGy).

The mean APEs of SNs at all dose settings were the lowest with ClariCT.AI, with a significant difference at 80 kVp/40 mA (CTDI_vol_, 0.2 mGy) (P<0.05).

The ICCs of both radiologists were excellent (ICC, 0.879–0.967).

### Mean APE according to SN size

Table 4 shows the APEs according to different-sized SNs with four different radiation dose settings. The mean APEs obtained with TFI and ClariCT.AI for all SNs measuring at least 5 mm (including 5-mm, 8-mm, and 10-mm SNs) at all dose settings were less than 10%.

**Table 4:** Absolute percentage measurement error according to solid nodule size.

Radiation dose (CTDIvol)	Nodule size (mm)	ASiR-V	TFI	ClariCT.AI	P-value
120 kVp/220 mA (3.39 mGy)	3	17.24 ± 6.03	14.59 ± 5.70	4.11 ± 4.13*	**0.024**
	5	5.58 ± 2.52	4.05 ± 1.57	1.68 ± 1.44	0.105
	8	5.41 ± 6.29	7.65 ± 4.87	4.76 ± 2.77	0.368
	10	9.72 ± 7.76	8.86 ± 6.40	8.72 ± 5.90	0.779
120 kVp/90 mA (1.39 mGy)	3	18.83 ± 6.20	13.88 ± 5.52	8.80 ± 5.34*	**0.018**
	5	3.25 ± 3.66	5.35 ± 3.24	4.28 ± 4.40	0.368
	8	11.27 ± 7.45	6.81 ± 5.32	3.45 ± 2.87	0.174
	10	4.09 ± 4.93	6.67 ± 4.28	9.20 ± 3.07	0.472
120 kVp/40 mA (0.62 mGy)	3	17.24 ± 5.48	6.42 ± 5.58*	6.90 ± 6.70*	**0.049**
	5	5.88 ± 3.26	3.02 ± 2.68	5.62 ± 2.49	0.174
	8	9.61 ± 4.56	5.13 ± 5.11	5.80 ± 3.54	0.174
	10	6.90 ± 6.89	7.01 ± 7.16	6.47 ± 3.14	>0.999
80 kVp/40 mA (0.2 mGy)	3	26.89 ± 6.80	17.59 ± 6.07*	17.96 ± 8.58	**0.038**
	5	5.08 ± 2.61	3.63 ± 2.11	1.95 ± 2.31	0.368
	8	15.40 ± 11.17	9.40 ± 10.79	5.61 ± 3.69	0.420
	10	9.44 ± 7.60	5.38 ± 6.68	5.90 ± 2.63	0.368

Note—CTDIvol, CT dose index volume.

*Comparison with ASiR-V, P<0.05

**Fig 1:**
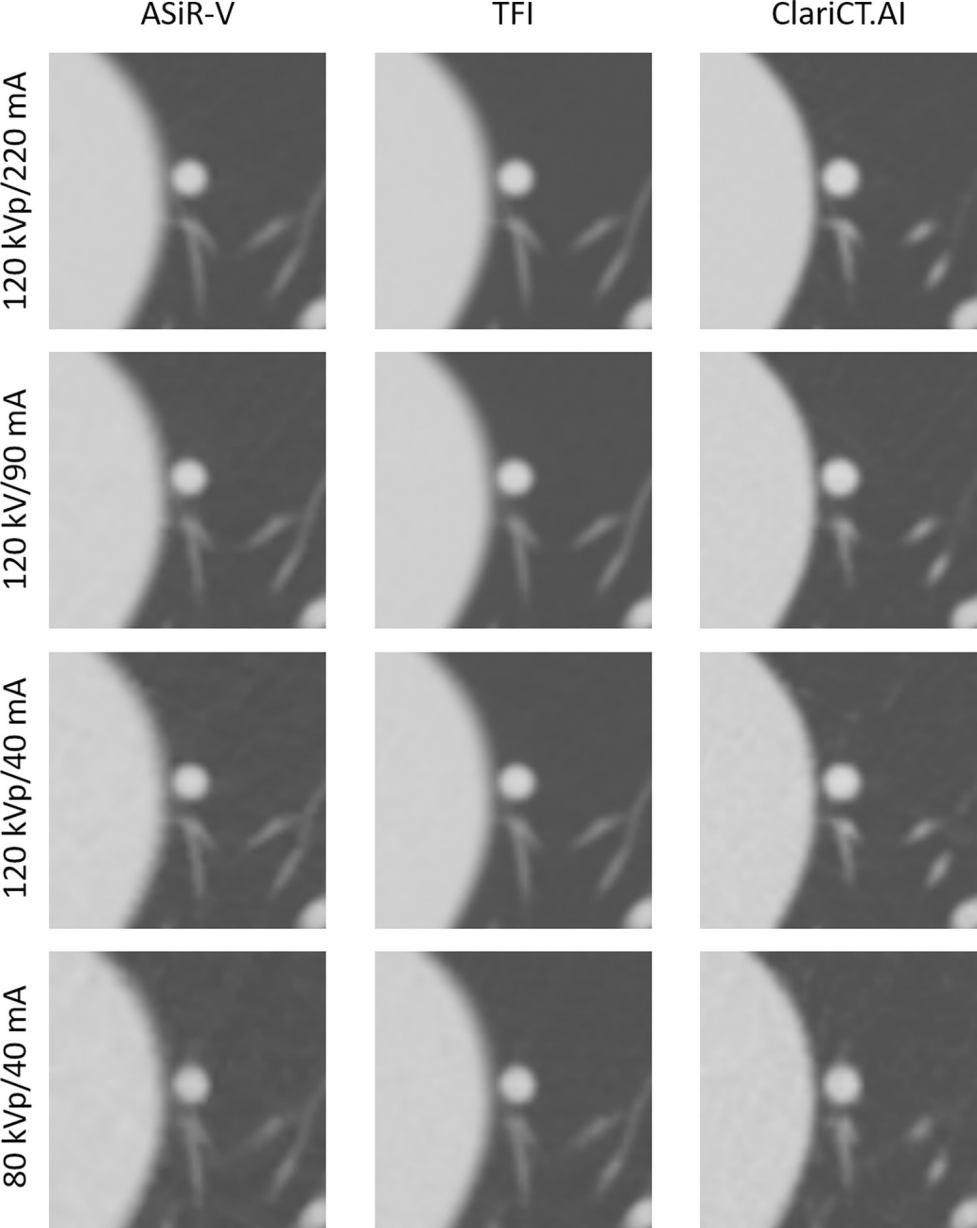
CT images of SNs at different radiation dose settings. Images of SNs (5 mm) at four radiation dose settings reconstructed with ASiR-V, TFI, and ClariCT.AI.

The lowest APEs were observed with the deep learning–based algorithms, TFI or ClariCT.AI (Fig 1); however, only the APEs for 3-mm SNs reconstructed with TFI or ClariCT.AI at each radiation dose setting yielded significant differences among the algorithms investigated. At 120 kVp/220 mA (CTDI_vol_, 3.39 mGy) and 120 kVp/90 mA (CTDI_vol_, 1.39 mGy), ClariCT.AI yielded the lowest APE among the algorithms, and at 120 kVp/40 mA (CTDI_vol_, 0.62 mGy) and 80 kVp/40 mA (CTDI_vol_, 0.2 mGy), TFI yielded the lowest APE among the algorithms; these results were statistically significant (all P<0.05). Additionally, TFI was associated with significantly lower APEs than ASiR-V at 120 kVp/40 mA (CTDI_vol_, 0.62 mGy) and 80 kVp/40 mA (CTDI_vol_, 0.2 mGy), and ClariCT.AI yielded significantly lower APEs than ASiR-V for 3-mm SNs at 120 kVp/220 mA (CTDI_vol_, 3.39 mGy), 120 kVp/90 mA (CTDI_vol_, 1.39 mGy), and 120 kVp/40 mA (CTDI_vol_, 0.62 mGy) (all P<0.05).

### Mean APE according to GGN size

Table 5 shows the APEs according to different GGN sizes at four different radiation dose settings. The mean APEs obtained with TFI for all GGNs at all dose settings were less than 10%, and the APEs for all GGNs evaluated with ClariCT.AI were less than 10% at 120 kVp/220 mA (CTDI_vol_, 3.39 mGy) and 120 kVp/90 mA (CTDI_vol_, 1.39 mGy).

**Table 5:** Absolute percentage measurement error according to nodule size and attenuation of ground-glass nodules.

Radiation dose (CTDIvol)	Attenuation of GGN	Nodule size (mm)	ASiR-V	TFI	ClariCT.AI	P-value
120 kVp/220 mA (3.39 mGy)	−630 HU	5	9.97 ± 5.59	2.25 ± 1.72	1.49 ± 1.50*	**0.023**
		8	2.36 ± 0.56	0.37 ± 0.04*	0.30 ± 0.18*	**0.032**
		10	2.36 ± 0.29	0.49 ± 0.45	0.93 ± 1.14	0.057
	−800 HU	5	19.02 ± 3.30	1.26 ± 0.40*	1.26 ± 0.89*	**0.032**
		8	0.95 ± 0.78	0.37 ± 0.40*	2.05 ± 2.04	0.319
		10	4.17 ± 1.61	1.36 ± 1.15	1.36 ± 1.74	0.058
120 kVp/90 mA (1.39 mGy)	−630 HU	5	16.31 ± 14.08	2.64 ± 1.50	8.98 ± 7.91	0.472
		8	7.21 ± 5.90	3.64 ± 2.38	5.02 ± 2.68	0.057
		10	6.37 ± 3.22	0.87 ± 0.25	1.50 ± 2.01	0.174
	−800 HU	5	28.23 ± 8.22	2.64 ± 2.25*	6.80 ± 3.30	**0.039**
		8	8.70 ± 3.94	3.47 ± 0.68	5.35 ± 1.84	0.368
		10	15.38 ± 10.43	4.34 ± 2.42	5.41 ± 2.13	0.472
120 kVp/40 mA (0.62 mGy)	−630 HU	5	22.35 ± 5.90	3.02 ± 1.99*	15.62 ± 0.08	**0.039**
		8	7.86 ± 0.30	1.50 ± 1.34*	4.13 ± 1.52	**0.018**
		10	10.48 ± 0.18	2.16 ± 1.26*	5.77 ± 2.47	**0.018**
	−800 HU	5	24.60 ± 0.89	4.51 ± 1.53*†	19.67 ± 13.10	**0.049**
		8	15.42 ± 0.36	3.57 ± 4.37*	6.28 ± 3.16	**0.038**
		10	15.49 ± 0.11	0.48 ± 0.29*	7.11 ± 5.15	**0.018**
80 kVp/40 mA (0.2 mGy)	-630 HU	5	15.66 ± 7.86	5.12 ± 3.85*	5.19 ± 6.03	**0.368**
		8	12.34 ± 4.82	6.61 ± 5.79*	9.06 ± 3.53	**0.031**
		10	18.93 ± 1.44	0.77 ± 0.72*	1.68 ± 1.08	**0.022**
	-800 HU	5	26.59 ± 7.03	4.47 ± 5.69*	13.90 ± 7.13	**0.039**
		8	19.99 ± 5.53	6.99 ± 4.20*	9.43 ± 2.61*	**0.049**
		10	31.15 ± 2.17	7.92 ± 7.18*	6.84 ± 4.07*	**0.049**

Note—CTDIvol, CT dose index volume, GGN, ground-glass nodule.

*Comparison with ASiR-V, P<0.05

†Comparison with ClariCT.AI, P<0.05

**Fig 2:**
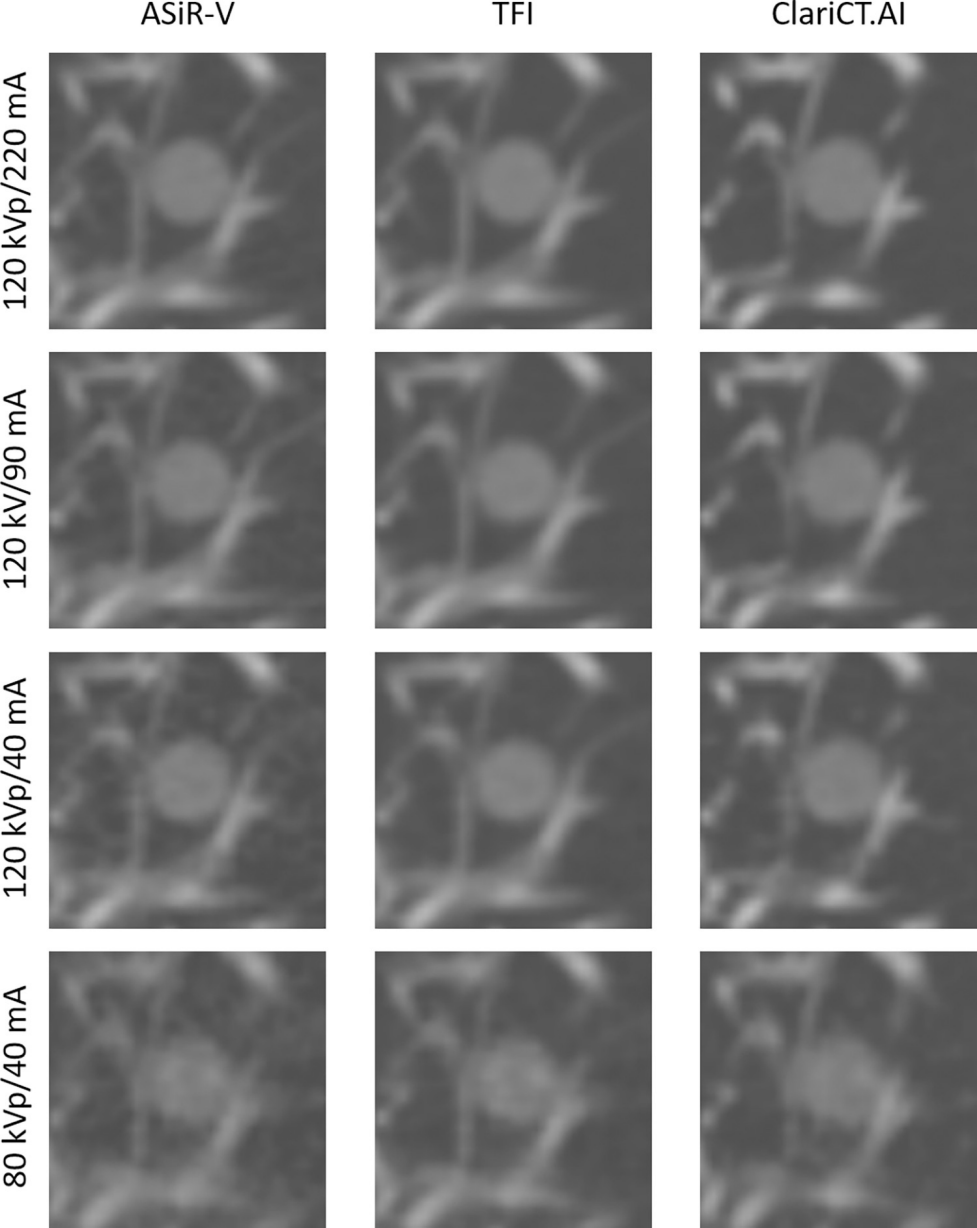
CT images of GGNs at different radiation dose settings. Images of GGNs (8 mm) with an attenuation of −630 HU at four radiation dose settings reconstructed with ASiR-V, TFI, and ClariCT.AI.

**Fig 3:**
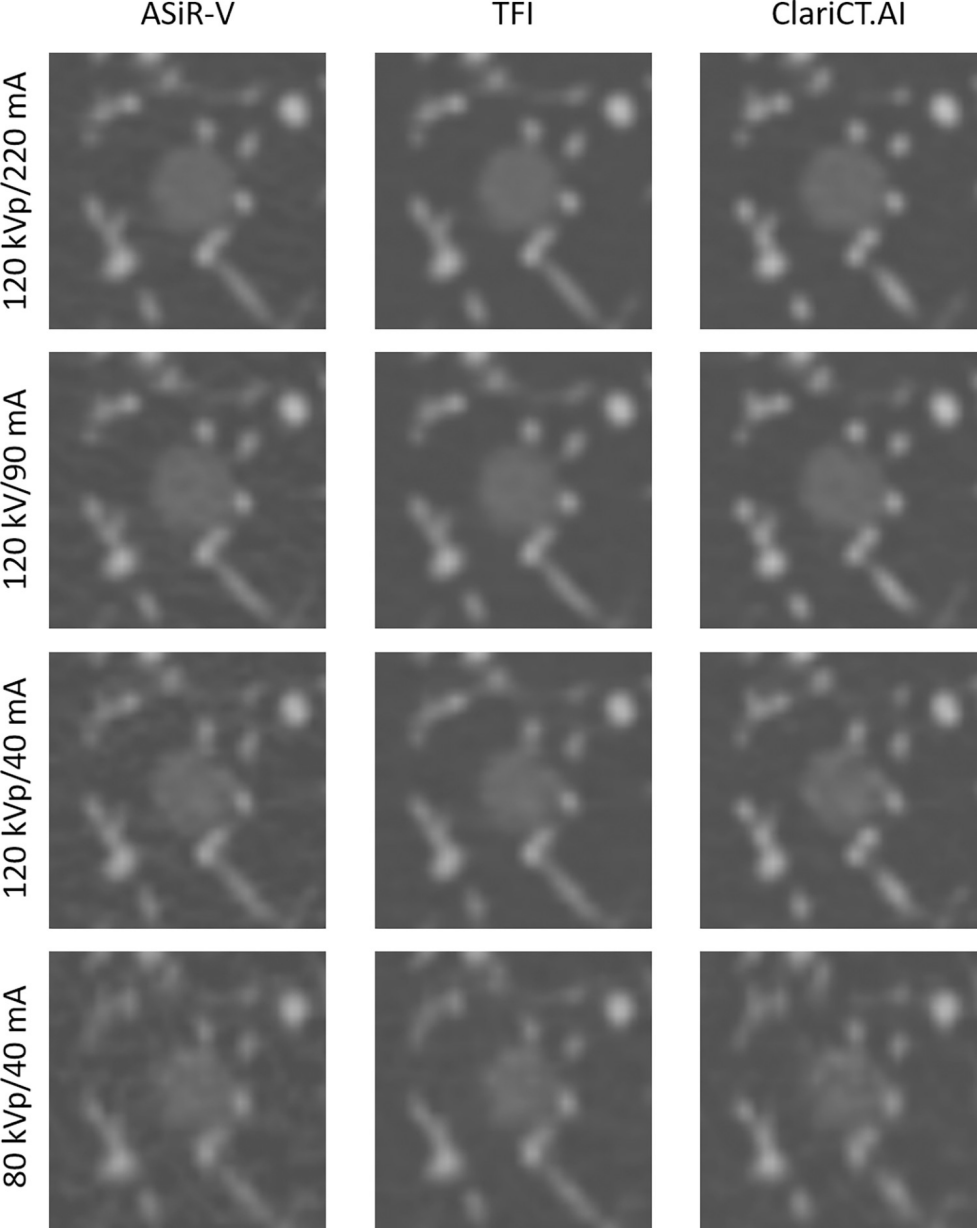
CT images of GGNs at different radiation dose settings. Images of GGNs (8 mm) with an attenuation of −800 HU at four radiation dose settings reconstructed with ASiR-V, TFI, and ClariCT.AI.

The lowest APEs of GGNs were observed with TFI or ClariCT.AI at all dose settings. Among the deep learning–based algorithms, TFI yielded the lowest APEs at 120 kVp/40 mA (CTDI_vol_, 0.62 mGy) and 80 kVp/40 mA for all sizes of GGNs of −630 and −800 HU (Figs 2 and 3), except for one 10-mm GGNs of −800 HU at 80 kVp/40 mA (all P<0.05). The APEs obtained with TFI at 120 kVp/40 mA (CTDI_vol_, 0.62 mGy) and 80 kVp/40 mA (CTDI_vol_, 0.2 mGy) were significantly lower than those obtained with ASiR-V for all sizes of GGNs of both −630 and −800 HU (all P<0.05).

### Image noise of the algorithms

The measured image noise values are reported in Table 6. Image noise was the lowest with TFI, and TFI was associated with significantly lower image noise than ASiR-V at all dose settings (all P<0.05). At 120 kVp/220 mA (CTDI_vol_, 3.39 mGy), 120 kVp/90 mA (CTDI_vol_, 1.39 mGy), and 120 kVp/40 mA (CTDI_vol_, 0.62 mGy), TFI yielded significantly lower image noise than ClariCT.AI (all P<0.05). At 120 kVp/90 mA (CTDI_vol_, 1.39 mGy), 120 kVp/40 mA (CTDI_vol_, 0.62 mGy), and 80 kVp/40 mA (CTDI_vol_, 0.2 mGy), image noise on CT scans reconstructed with ClariCT.AI was significantly lower than image noise on CT scans reconstructed with ASiR-V (all P<0.05).

**Table 6:** Comparison of image noise values of different algorithms.

Radiation dose (CTDI_vol_)	ASiR-V	TFI	ClariCT.AI	P-value
120 kVp/220 mA (3.39 mGy)	7.95±1.32	3.34±0.47*†	6.56±0.53	0.002
120 kVp/90 mA (1.39 mGy)	11.48±1.44	4.65±0.92*†	7.71±0.96*	0.001
120 kVp/40 mA (0.62 mGy)	16.69±0.63	6.71±0.53*†	12.16±1.37*	<0.001
80 kVp/40 mA (0.2 mGy)	18.73±2.83	10.73±1.83*	13.15±2.21*	0.014

Note—CTDI_vol_, CT dose index volume

*Comparison with ASiR-V, P<0.05

†Comparison with ClariCT.AI, P<0.05

### TTF and NPS analysis

The results of TTF curves for all reconstruction type with the bone and acrylic inserts are shown in S1 and S2 Figs. S1 Table reports the TTF_50%_ values for both inserts, and TTF_50%_ values tended to decrease as dose decreased. For the bone insert, TTF_50%_ values of TFI and ClariCT.AI were higher than those of ASiR-V at all dose settings. For the acrylic insert, TFI yielded the highest TTF_50%_ values at all dose settings.

S2 Table reports the NPS peak and average spatial frequency data and S3 Fig. shows the NPS curves for all reconstruction types and all dose settings. The NPS peak increased as the dose decreased. NPS peaks were lower with TFI and ClariCT.AI than ASiR-V, and TFI yielded the lowest NPS peak at all dose settings among the reconstruction algorithms. In all dose conditions, ClariCT.AI shifted toward a lower average spatial frequency compared with ASiR-V, but TFI yielded a similar level to that of ASiR-V.

## Discussion

Our study demonstrated that deep learning–based algorithms were more appropriate for measuring the volumes of both SNs and GGNs than ASiR-V using both low-dose and ultra–low-dose settings. The volumetric measurement errors associated with TFI and ClariCT.AI across all nodules at all dose settings were less than 11%. Specifically, the volumes of the smallest SNs (3 mm) at all radiation dose settings and the volumes of all GGNs, including the smallest GGNs (5 mm) and low-attenuation GGNs (−800 HU), at ultra–low-dose settings less than 1 mSv were most accurately measured when TFI was used for reconstruction. TFI was also superior to ASiR-V in terms of image noise and spatial resolution.

Although recent studies have revealed improved image quality or increased lesion detectability with deep learning image reconstruction compared with IR, no published studies have evaluated the accuracy of pulmonary nodule volume measurement [6, 7]. Greffier et al. performed a phantom study and demonstrated increased detectability with TFI relative to ASiR-V, permitting dose reduction [7]. However, the lesions in the phantom were not pulmonary nodules but a large mass in the liver, a small calcification, and a small subtle lesion with low contrast. The study performed by Kim et al. showed that TFI was superior to ASiR-V for identifying anatomic structures in the human thorax, such as the pulmonary arteries/veins, trachea/bronchi, lymph nodes, and pleura/pericardium, and had an increased SNR and CNR, but they did not analyze detectability or volumetry of lung nodules [11]. Hata et al. found that TFI was associated with significantly less noise, a higher SNR/CNR, and finer image texture than ASiR-V [9]. They also demonstrated that the combination of deep learning–based denoising and IR improved image quality and Lung Imaging Reporting and Data System (Lung-RADS) evaluation on ultra–low-dose CT [18].

Due to technical factors affecting nodule volumetry measurement reliability (e.g., interobserver variability between radiologists, software packages, CT manufacturers, intravenous contrast material, inspiratory effort), approximately 5–25% variation has been reported between different CT scans of the same nodule performed on the same day [24]. Therefore, a volume change <25% may be due to interscan variability and is considered “absence of nodule growth”, whereas a volume change >100% usually indicates obvious nodule growth [24]. Therefore, it might be acceptable that the volumetric measurement errors of TFI and ClariCT.AI across all nodules at all dose settings were less than 11% in our study results. In general, except for some cases, the smaller the size, the larger the volumetric measurement error at all dose settings for both SNs and GGNs. For example, the mean APEs of 3-mm SNs at 80 kVp/40 mA (CTDI_vol_, 0.2 mGy) were 26.89 ± 6.80 for ASiR-V, 17.59 ± 6.07 with TFI, and 17.96 ± 8.58 with ClariCT.AI, whereas the mean APEs of 10-mm SNs were 9.44 ± 7.60 with ASiR-V, 5.38 ± 6.68 with TFI, and 5.90 ± 2.63 with ClariCT.AI, suggesting that the smaller the nodule size, the more careful the interpretation of the results of volumetric measurement is. However, in our study, among all APEs of SNs and GGNs, only some ASiR-V instances yielded volumetric measurement errors of 25% or more.

The present study showed significantly improved accuracy of volumetry of SNs and GGNs using TFI or ClariCT.AI compared with ASiR. Additionally, for 5-mm, 8-mm, and 10-mm SNs, the volumetric measurement errors of TFI and ClariCT.AI in low-dose and ultra–low-dose settings were less than 10%. For a 3-mm SN, the volumetric measurement errors of TFI were less than 20%, and those of ClariCT.AI were less than 10% except in association with a dose setting of 80 kVp/40 mA (CTDI_vol_, 0.2 mGy); however, TFI yielded a significantly lower APE than ASiR-V (P<0.05). Therefore, ultra–low-dose CT scans of less than 1 mSv reconstructed with TFI or ClariCT.AI could be used for follow-up of SNs measuring at least 5 mm, and scans obtained at a dose setting of at least 120 kVp/40mA (CTDI_vol_, 0.62 mGy) and reconstructed with TFI or ClariCT.AI might be acceptable for follow-up of SNs measuring 3 mm.

For GGN volumetry, we used two types of GGNs with different attenuations (−630 HU and −800 HU). For all reconstructions at all dose settings, the measurement error tended to be higher for −800 HU GGNs than for −630 HU GGNs, probably because −800 HU GGNs are fainter. However, the volumetric measurement errors associated with all sizes of GGNs at all dose settings assessed with TFI were less than 10%. Additionally, at dose settings of 120 kVp/220 mA (CTDI_vol_, 3.39 mGy) and 120 kVp/90 mA (CTDI_vol_, 1.39 mGy), the volumetric measurement errors for all sizes of GGNs evaluated with ClariCT.AI were less than 10%. TFI and ClariCT.AI yielded significantly lower APEs at 120 kVp/40 mA (CTDI_vol_, 0.62 mGy) and 80 kVp/40 mA (CTDI_vol_, 0.2 mGy) for all sizes of −630 and −800 HU GGNs. For follow-up of GGNs measuring at least 5 mm and −800 HU, ultra–low-dose CT scans performed at less than 1 mSv and reconstructed with TFI could be useful, and low-dose CT scans performed using dose settings of at least 120 kVp/90 mA (CTDI_vol_, 1.39 mGy) and reconstructed with ClariCT.AI might be acceptable.

We also found that significant image noise reduction with TFI and ClariCT.AI compared with ASiR-V. TFI was associated with the lowest image noise among the algorithms at all dose settings, including on ultra–low-dose chest CT scans performed at less than 1 mSv; these results aligned with those of a previous study [7]. TFI yielded significantly lower image noise than ClariCT.AI at 120 kVp/220 mA (CTDI_vol_, 3.39 mGy), 120 kVp/90 mA (CTDI_vol_, 1.39 mGy), and 120 kVp/40 mA (CTDI_vol_, 0.62 mGy). At 120 kVp/90 mA (CTDI_vol_, 1.39 mGy) and 120 kVp/40 mA (CTDI_vol_, 0.62 mGy), ClariCT.AI also yielded significantly lower image noise than ASiR-V.

TTF is a representative metric of spatial resolution, and the TTF curve provides the degree of contrast ratio of the original object across spatial frequencies [25]. Therefore, a higher TTF value means better spatial resolution. In our study, for the bone insert, higher TTF_50%_ values were associated with TFI and ClariCT.AI than with ASiR-V at all dose settings, and the highest TTF_50%_ values were also associated with in TFI among the reconstruction algorithms at all dose settings, indicative of the better spatial resolution of the deep learning-based algorithms. NPS is a method that can determine the amount of noise (magnitude) and noise characteristics (texture) in the spatial frequency domain [26]. This is a way to overcome the drawback of pixel SD in evaluating image noise, and is commonly used for image quality evaluation, optimization, and image-to-image comparison. In our study, NPS peaks were lower with TFI and ClariCT.AI than with ASiR-V, and TFI yielded the lowest NPS peak at all dose settings among the reconstruction algorithms, maintaining textures. Similar results have also been reported in previous studies. Greffier et al. assessed the impact on image quality and radiation dose of TFI (compared with a hybrid IR algorithm) using TTF and NPS, and they demonstrated that TFI reduced noise and improved spatial resolution and detectability without perceived alteration of the texture, similar to that of conventional IR [7]. Also, ClariCT.AI improved spatial resolution characteristics and slightly lowered noise compared with conventional IR [27]. Based on these results, improved accuracy of nodule volumetry might be possible with the deep learning-based reconstruction algorithms highlighted by our study.

TFI is a deep learning–based reconstruction algorithm developed by a specific CT machine manufacturer (GE Healthcare). Currently, TFI can only be applied to a specific CT scanner made by GE Healthcare. On the other hand, ClariCT.AI is a deep learning–based denoising software that has the advantage of being applicable to images obtained by all CT scanners. In this study, the accuracy of lung nodule volumetry with TFI and ClariCT.AI was significantly superior to that assessed with IR, but TFI could be applied at lower radiation doses than those compatible with ClariCT.AI for follow-up of 5-mm GGNs. TFI was also associated with significantly lower image noise than ClariCT.AI at 120 kVp/90 mA (CTDI_vol_, 1.39 mGy) and 120 kVp/40 mA (CTDI_vol_, 0.62 mGy). However, despite these limitations, ClariCT.AI was also sufficiently accurate for nodule volumetry in low-dose and ultra–low-dose settings, and research including images obtained by scanners from other vendors is needed.

There were several limitations to our study. First, we did not evaluate 3-mm GGNs because we did not have any available to us. However, according to lung nodule management guidelines from the Fleischner Society, no routine follow-up is generally recommended for pure GGNs measuring 6 mm or less in diameter. Furthermore, we demonstrated results using 5-mm GGNs for volumetric accuracy. Second, the lack of real lung parenchyma in the chest phantom may have contributed to increased measurement reproducibility. Third, only 1.25-mm slice thicknesses and “Lung” kernel for reconstruction were used in this study; therefore, the generalizability of our results to other slice thickness or reconstruction kernels could not be studied. We used 1.25-mm slice thicknesses and the “Lung” kernel for reconstruction for all CT image datasets because these conditions are recommended by Korean Lung Cancer Screening Project (K-LUCAS) [28]. As the purpose of the present study was to compare the accuracy of two deep learning-based algorithms with IR for volume measurement of SNs and GGNs of various sizes at low-dose and ultra-low-dose settings, the CT protocol (1.25-mm slice thickness and “Lung” kernel for reconstruction) remained unchanged. However, further research on this topic is warranted.

## Conclusion

In conclusion, TFI and ClariCT.AI were more accurate than ASiR-V for obtaining volume measurements of both SNs and GGNs using low-dose and ultra–low-dose settings. The volumetric measurement errors associated with TFI and ClariCT.AI across all nodules at all dose settings were less than 11%. Ultra–low-dose CT scans reconstructed with TFI or ClariCT.AI could be used for follow-up of SNs measuring at least 5 mm. For follow-up of GGNs measuring at least 5 mm and −800 HU, ultra–low-dose CT performed at less than 1 mSv and reconstructed with TFI could be useful, and low-dose CT performed at dose settings of least 120 kVp/90mA (CTDI_vol_, 1.39 mGy) and reconstructed with ClariCT.AI might be acceptable. TFI was also superior to ASiR-V with regard to image noise when using ultra–low-dose CT.

## Supporting information

S1 Fig The results of TTF curves measured in bone insert at each dose setting. (A) 120 kVp/220 mA, (B) 120 kVp/90 mA, (C) 120 kVp/40 mA, (D) 80 kVp/40 mA.
(DOCX)

S2 Fig The results of TTF curves measured in acrylic insert at each dose setting. (A) 120 kVp/220 mA, (B) 120 kVp/90 mA, (C) 120 kVp/40 mA, (D) 80 kVp/40 mA.
(DOCX)

S3 Fig The results of NPS curves at each dose setting. (A) 120 kVp/220 mA, (B) 120 kVp/90 mA, (C) 120 kVp/40 mA, (D) 80 kVp/40 mA.
(DOCX)

S1 Table The results of TTF_50%_ for bone and acrylic inserts. (DOCX)

S2 Table The NPS peak and average spatial frequency data at each dose settings. (DOCX)

## References

[1] Abraham JJCO. Reduced lung cancer mortality with low-dose computed tomographic screening. 2011;8(10):441–2. 10.1056/NEJMoa1102873 PMC4356534 21714641

[2] MacMahon H, Naidich DP, Goo JM, Lee KS, Leung ANC, Mayo JR, et al. Guidelines for Management of Incidental Pulmonary Nodules Detected on CT Images: From the Fleischner Society 2017. Radiology. 2017;284(1):228–43. Epub 2017/02/28. doi: 10.1148/radiol.2017161659 . 28240562

[3] Kim SK, Kim C, Lee KY, Cha J, Lim HJ, Kang EY, et al. Accuracy of Model-Based Iterative Reconstruction for CT Volumetry of Part-Solid Nodules and Solid Nodules in Comparison with Filtered Back Projection and Hybrid Iterative Reconstruction at Various Dose Settings: An Anthropomorphic Chest Phantom Study. Korean journal of radiology. 2019;20(7):1195–206. Epub 2019/07/05. doi: 10.3348/kjr.2018.0893 ; PubMed Central PMCID: PMC6609437. 31270983 PMC6609437

[4] Kim H, Park CM, Song YS, Lee SM, Goo JM. Influence of radiation dose and iterative reconstruction algorithms for measurement accuracy and reproducibility of pulmonary nodule volumetry: A phantom study. European journal of radiology. 2014;83(5):848–57. Epub 2014/02/28. doi: 10.1016/j.ejrad.2014.01.025 . 24572380

[5] Kim H, Park CM, Kim SH, Lee SM, Park SJ, Lee KH, et al. Persistent pulmonary subsolid nodules: model-based iterative reconstruction for nodule classification and measurement variability on low-dose CT. European radiology. 2014;24(11):2700–8. Epub 2014/07/21. doi: 10.1007/s00330-014-3306-7 . 25038857

[6] Park C, Choo KS, Jung Y, Jeong HS, Hwang JY, Yun MS. CT iterative vs deep learning reconstruction: comparison of noise and sharpness. European radiology. 2020. Epub 2020/10/16. doi: 10.1007/s00330-020-07358-8 . 33057781

[7] Greffier J, Hamard A, Pereira F, Barrau C, Pasquier H, Beregi JP, et al. Image quality and dose reduction opportunity of deep learning image reconstruction algorithm for CT: a phantom study. European radiology. 2020;30(7):3951–9. Epub 2020/02/27. doi: 10.1007/s00330-020-06724-w . 32100091

[8] Sammut B, Tran R, Okerlund DJJoCCT. Application Of Deep Learning Image Reconstruction For Coronary Artery Calcium Scoring. 2020;14(3):S18–S9.

[9] Hata A, Yanagawa M, Yoshida Y, Miyata T, Kikuchi N, Honda O, et al. The image quality of deep-learning image reconstruction of chest CT images on a mediastinal window setting. Clinical radiology. 2020. Epub 2020/11/23. doi: 10.1016/j.crad.2020.10.011 . 33220941

[10] Kim I, Kang H, Yoon HJ, Chung BM, Shin NY. Deep learning-based image reconstruction for brain CT: improved image quality compared with adaptive statistical iterative reconstruction-Veo (ASIR-V). Neuroradiology. 2020. Epub 2020/10/11. doi: 10.1007/s00234-020-02574-x . 33037503

[11] Kim JH, Yoon HJ, Lee E, Kim I, Cha YK, Bak SH. Validation of Deep-Learning Image Reconstruction for Low-Dose Chest Computed Tomography Scan: Emphasis on Image Quality and Noise. Korean journal of radiology. 2020. Epub 2020/07/31. doi: 10.3348/kjr.2020.0116 . 32729277 PMC7772377

[12] Benz DC, Benetos G, Rampidis G, von Felten E, Bakula A, Sustar A, et al. Validation of deep-learning image reconstruction for coronary computed tomography angiography: Impact on noise, image quality and diagnostic accuracy. Journal of cardiovascular computed tomography. 2020;14(5):444–51. Epub 2020/01/25. doi: 10.1016/j.jcct.2020.01.002 . 31974008

[13] Cao L, Liu X, Li J, Qu T, Chen L, Cheng Y, et al. A study of using a deep learning image reconstruction to improve the image quality of extremely low-dose contrast-enhanced abdominal CT for patients with hepatic lesions. The British journal of radiology. 2020:20201086. Epub 2020/11/27. doi: 10.1259/bjr.20201086 . 33242256 PMC7934287

[14] Solomon J, Lyu P, Marin D, Samei E. Noise and spatial resolution properties of a commercially available deep learning-based CT reconstruction algorithm. Medical physics. 2020;47(9):3961–71. Epub 2020/06/09. doi: 10.1002/mp.14319 . 32506661

[15] Jensen CT, Liu X, Tamm EP, Chandler AG, Sun J, Morani AC, et al. Image Quality Assessment of Abdominal CT by Use of New Deep Learning Image Reconstruction: Initial Experience. AJR American journal of roentgenology. 2020;215(1):50–7. Epub 2020/04/15. doi: 10.2214/AJR.19.22332 . 32286872

[16] Hong JH, Park EA, Lee W, Ahn C, Kim JH. Incremental Image Noise Reduction in Coronary CT Angiography Using a Deep Learning-Based Technique with Iterative Reconstruction. Korean journal of radiology. 2020;21(10):1165–77. Epub 2020/07/31. doi: 10.3348/kjr.2020.0020 ; PubMed Central PMCID: PMC7458859. 32729262 PMC7458859

[17] Singh R, Digumarthy SR, Muse VV, Kambadakone AR, Blake MA, Tabari A, et al. Image Quality and Lesion Detection on Deep Learning Reconstruction and Iterative Reconstruction of Submillisievert Chest and Abdominal CT. AJR American journal of roentgenology. 2020;214(3):566–73. Epub 2020/01/23. doi: 10.2214/AJR.19.21809 . 31967501

[18] Hata A, Yanagawa M, Yoshida Y, Miyata T, Tsubamoto M, Honda O, et al. Combination of Deep Learning-Based Denoising and Iterative Reconstruction for Ultra-Low-Dose CT of the Chest: Image Quality and Lung-RADS Evaluation. AJR American journal of roentgenology. 2020;215(6):1321–8. Epub 2020/10/15. doi: 10.2214/AJR.19.22680 . 33052702

[19] Hein PA, Romano VC, Rogalla P, Klessen C, Lembcke A, Dicken V, et al. Linear and volume measurements of pulmonary nodules at different CT dose levels—intrascan and interscan analysis. RoFo: Fortschritte auf dem Gebiete der Rontgenstrahlen und der Nuklearmedizin. 2009;181(1):24–31. Epub 2008/12/17. doi: 10.1055/s-2008-1027874 . 19085687

[20] Medicine AAoPi. The measurement, reporting, and management of radiation dose in CT. AAPM report; 2008.

[21] Kim C, Lee SM, Choe J, Chae EJ, Do K-H, Seo JB. Volume doubling time of lung cancer detected in idiopathic interstitial pneumonia: comparison with that in chronic obstructive pulmonary disease. European radiology. 2018;28(4):1402–9. doi: 10.1007/s00330-017-5091-6 29038933

[22] Kim C, Lee KY, Shin C, Kang E-Y, Oh Y-W, Ha M, et al. Comparison of filtered back projection, hybrid iterative reconstruction, model-based iterative reconstruction, and virtual monoenergetic reconstruction images at both low-and standard-dose settings in measurement of emphysema volume and airway wall thickness: a CT phantom study. Korean journal of radiology. 2018;19(4):809–17. doi: 10.3348/kjr.2018.19.4.809 29962888 PMC6005943

[23] Friedman SN, Fung GS, Siewerdsen JH, Tsui BM. A simple approach to measure computed tomography (CT) modulation transfer function (MTF) and noise-power spectrum (NPS) using the American College of Radiology (ACR) accreditation phantom. Medical physics. 2013;40(5):051907. doi: 10.1118/1.4800795 ; PubMed Central PMCID: PMC3643984. 23635277 PMC3643984

[24] Devaraj A, van Ginneken B, Nair A, Baldwin D. Use of Volumetry for Lung Nodule Management: Theory and Practice. Radiology. 2017;284(3):630–44. Epub 2017/08/22. doi: 10.1148/radiol.2017151022 . 28825886

[25] Richard S, Husarik DB, Yadava G, Murphy SN, Samei E. Towards task-based assessment of CT performance: system and object MTF across different reconstruction algorithms. Medical physics. 2012;39(7):4115–22. doi: 10.1118/1.4725171 . 22830744

[26] Kijewski MF, Judy PF. The noise power spectrum of CT images. Physics in Medicine & Biology. 1987;32(5):565. doi: 10.1088/0031-9155/32/5/003 3588670

[27] Lim WH, Choi YH, Park JE, Cho YJ, Lee S, Cheon JE, et al. Application of Vendor-Neutral Iterative Reconstruction Technique to Pediatric Abdominal Computed Tomography. Korean journal of radiology. 2019;20(9):1358–67. doi: 10.3348/kjr.2018.0715 ; PubMed Central PMCID: PMC6715563. 31464114 PMC6715563

[28] Kim HY. National lung cancer screening in Korea: introduction and imaging quality control. Journal of the Korean Society of Radiology. 2019;80(5):826–36.

