# Supplementary figures and images for: Accuracy of two deep learning–based reconstruction methods compared with an adaptive statistical iterative reconstruction method for solid and ground-glass nodule volumetry on low-dose and ultra–low-dose chest computed tomography: A phantom study
Source: PLoS One. 2022 Jun 23;17(6):e0270122. doi: 10.1371/journal.pone.0270122 (PMC9223620; doi:10.1371/journal.pone.0270122)

**S3 Figure. The results of NPS curves at each dose setting.** (A) 120 kVp/220 mA, (B) 120 kVp/90 mA, (C) 120 kVp/40 mA, (D) 80 kVp/40 mA.

(A)


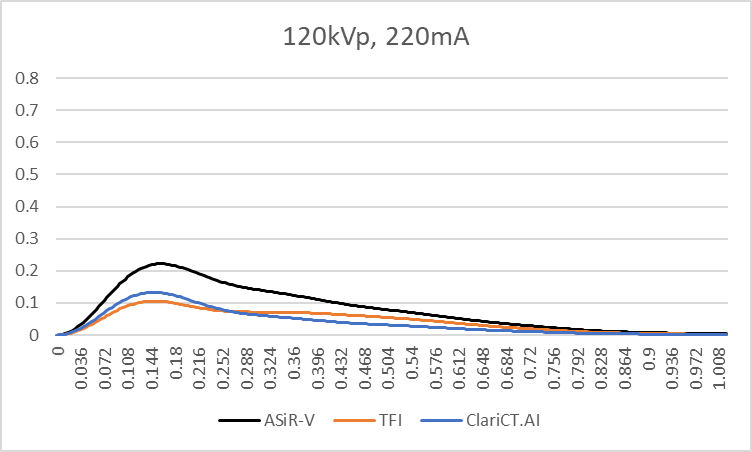


(B)


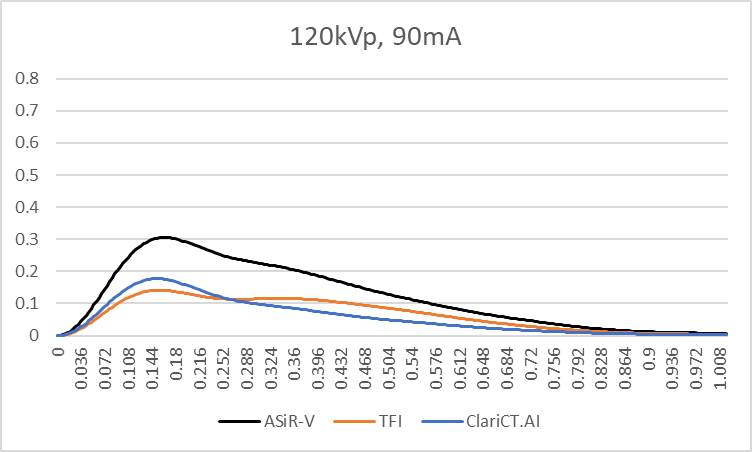


(C)


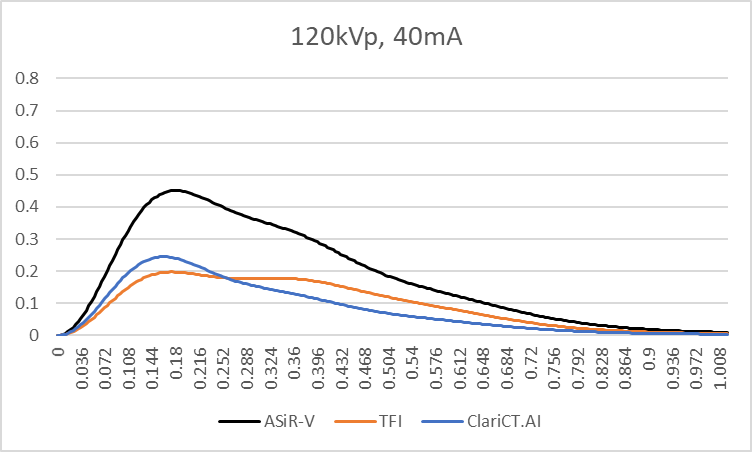


(D)


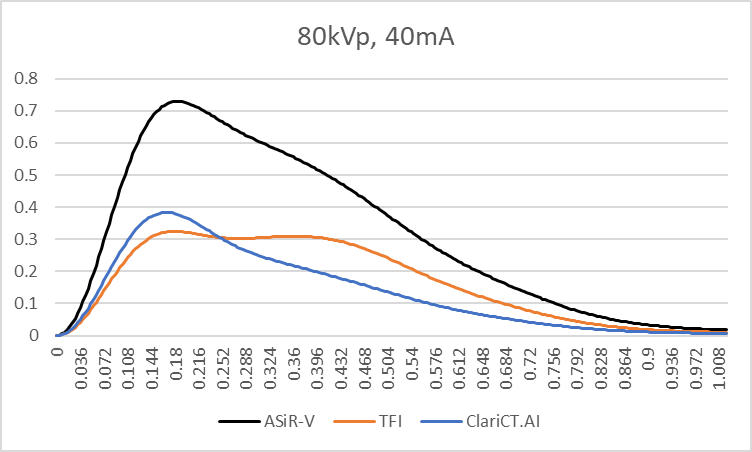

Supplement: S3 Fig — (A) 120 kVp/220 mA, (B) 120 kVp/90 mA, (C) 120 kVp/40 mA, (D) 80 kVp/40 mA. (DOCX) [file pone.0270122.s003.docx]
