# Supplementary material for: Accuracy of two deep learning–based reconstruction methods compared with an adaptive statistical iterative reconstruction method for solid and ground-glass nodule volumetry on low-dose and ultra–low-dose chest computed tomography: A phantom study
Source: PLoS One. 2022 Jun 23;17(6):e0270122. doi: 10.1371/journal.pone.0270122 (PMC9223620; doi:10.1371/journal.pone.0270122)
Supplement: S1 Table — (DOCX) [file pone.0270122.s004.docx]

**S1 Table. The results of TTF_50%_ for bone and acrylic inserts.**

| **kVp** | **mA** | **CTDI_vol_**  **(mGy)** | **TTF_50%_ (mm^-1^) bone insert** | | | **TTF_50%_ (mm^-1^) acrylic insert** | | |
| --- | --- | --- | --- | --- | --- | --- | --- | --- |
|  |  |  | **ASiR-V^*^** | **TFI^†^** | **ClariCT.AI** | **ASiR-V** | **TFI** | **ClariCT.AI** |
| 120 | 220 | 3.39 | 0.43 | 0.45 | 0.45 | 0.41 | 0.42 | 0.40 |
|  | 90 | 1.39 | 0.42 | 0.44 | 0.45 | 0.35 | 0.41 | 0.38 |
|  | 40 | 0.62 | 0.42 | 0.44 | 0.44 | 0.36 | 0.44 | 0.42 |
| 80 | 40 | 0.2 | 0.38 | 0.39 | 0.40 | 0.35 | 0.40 | 0.34 |

Note— CTDI_vol_, CT dose index volume.
