# Supplementary material for: Accuracy of two deep learning–based reconstruction methods compared with an adaptive statistical iterative reconstruction method for solid and ground-glass nodule volumetry on low-dose and ultra–low-dose chest computed tomography: A phantom study
Source: PLoS One. 2022 Jun 23;17(6):e0270122. doi: 10.1371/journal.pone.0270122 (PMC9223620; doi:10.1371/journal.pone.0270122)
Supplement: S2 Table — (DOCX) [file pone.0270122.s005.docx]

**S2 Table. The NPS peak and average spatial frequency data at each dose settings.**

| **kVp** | **mA** | **CTDI_vol_**  **(mGy)** | **NPS peak (HU^2^ mm^2^)** | | | **NPS average spatial frequency (mm^-1^)** | | |
| --- | --- | --- | --- | --- | --- | --- | --- | --- |
|  |  |  | **ASiR-V** | **TFI** | **ClariCT.AI** | **ASiR-V** | **TFI** | **ClariCT.AI** |
| 120 | 220 | 3.39 | 0.22 | 0.11 | 0.13 | 0.31 | 0.30 | 0.27 |
|  | 90 | 1.39 | 0.30 | 0.14 | 0.18 | 0.29 | 0.29 | 0.26 |
|  | 40 | 0.62 | 0.45 | 0.20 | 0.25 | 0.29 | 0.29 | 0.25 |
| 80 | 40 | 0.2 | 0.73 | 0.32 | 0.38 | 0.27 | 0.28 | 0.24 |

Note— CTDI_vol_, CT dose index volume.
